# Standardized construction of a porcine model for acute obstructive jaundice and training of endoscopic ultrasound-guided choledochoduodenostomy

**DOI:** 10.3389/fonc.2023.1247763

**Published:** 2023-11-21

**Authors:** Xue Zhou, Ziming Liu, Wenzhuang Ma, Shiyun Sheng, Siyu Sun, Nan Ge

**Affiliations:** Department of Gastroenterology, Shengjing Hospital of China Medical University, Shenyang, China

**Keywords:** biliary drainage, endoscopic ultrasound-guided choledochoduodenostomy, common bile duct, porcine model, stent

## Abstract

**Background:**

Endoscopic ultrasound-guided biliary drainage (EUS-BD) is an alternative to endoscopic retrograde cholangiopancreatography (ERCP) for patients with obstructive jaundice. However, it is still a challenge for many endoscopists because of its novelty and complexity. This study aimed to establish an ideal bile duct dilatation model for the training and practice of endoscopic ultrasound-guided choledochoduodenostomy (EUS-CDS).

**Methods:**

The 34 healthy Bama miniature pigs were divided into the part of developing the standardized EUS-CDS (n=9) and the part of trainees training (n=25). Part one, two different methods were used to clip Vater’s ampulla using metal clips to establish an extrahepatic bile duct dilatation model. Part two, five trainees were trained on EUS-CDS with 25 pigs. Following a 2-week observation period, the feasibility and effectiveness of the technique were evaluated.

**Results:**

In the group with three metal clips perpendicular to the duodenal wall clipping Vater’s ampulla, the success rate of extrahepatic bile duct dilation greater than 1 cm in 24 h was 5/6, whereas the remaining one pig was 48 h. All five trainees can finally complete the EUS-CDS independently. No death occurred during the 2-week observation period.

**Conclusion:**

Clipping Vater’s ampulla with three metal clips perpendicular to the duodenal wall is an effective and stable method to create a porcine bile duct dilatation model.

## 1 Introduction

Endoscopic retrograde cholangiopancreatography (ERCP) combined with biliary stent implantation is the standard palliative treatment for distal malignant biliary obstruction, but the failure rate is still 5–10% (1, 2). Since its introduction in 2001, endoscopic ultrasound-guided biliary drainage (EUS-BD) has gradually become the most preferred alternative drainage method after ERCP failure (3). However, EUS-BD is one of the most challenging endoscopic ultrasound interventional techniques for endoscopists, requiring a continuous learning cycle (4). Even in centers with a large number of biliary interventions, there are still not enough patients for young endoscopists under training (5).

Miniature pigs have been described as one of the most realistic animal models for EUS training, providing an anatomical structure similar to that of the human body and excellent tactile sensation (6, 7). Previous studies on the obstructive jaundice model focused mainly on surgical common bile duct (CBD) ligation, but this method is relatively more traumatic and unsuitable for endoscopic operation (8, 9). With the development of minimally invasive surgery in recent years, some scholars have used metal clips to clip Vater’s ampulla to construct a bile duct dilatation model (10). However, there are contrasting reports on this method because there are no detailed surgical procedures and stable results.

This study aimed to define the standard procedures for constructing a simple, reproducible, and stable extrahepatic bile duct dilatation model and verify its training effect among endoscopists who are proficient in EUS interventional treatment.

## 2 Materials and methods

### 2.1 Animals

**Figure 1:**
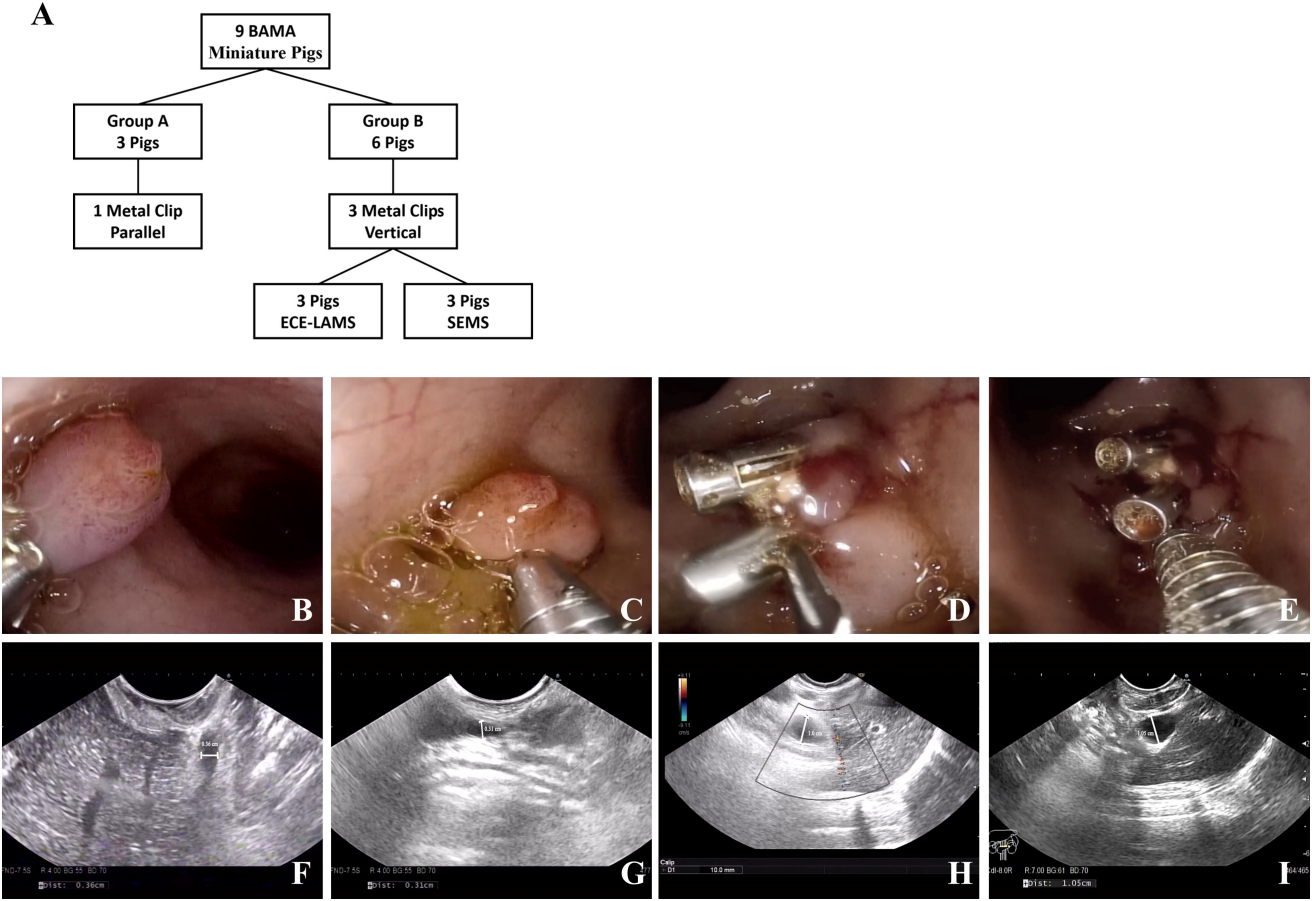
Experimental animal group design and model establishing. **(A)** The group design of experimental animals. **(B, C)** One metal clip parallel to the duodenum wall. **(D, E)** Three metal clips vertical to the duodenum wall. **(F, G)** No extrahepatic bile duct dilatation was observed in the control group under EUS (0.36/0.31 cm). **(H, I)** Extrahepatic bile duct dilatation was observed in the experimental group under EUS (1.0/1.05 cm).

In this study, 34 healthy Bama miniature male pigs (4–6 months, 10–15 kg) were used. Nine pigs were used to develop the standardized EUS-CDS process and the other twenty-five pigs were used for trainees training. Before the operation, the pigs were fasted for 24 h and given a full liquid diet. The experiment was conducted at the Endoscopy Center and the Experimental Animal Center of Shengjing Hospital of China Medical University. In standardized EUS-CDS process construction, the experimental animals were divided into a control group (Group A) with three pigs and two experimental groups (Group B) with three pigs each. In Group A, pigs had one metal clip placed parallel to the duodenal wall, whereas in Group B, three metal clips were placed perpendicular to the duodenal wall. Three pigs each from Group B were fitted with an electrocautery-enhanced lumen-apposing metal stent (ECE-LAMS) and a fully covered self-expandable metal stent (SEMS) (**Figure 1A**). During the training of the trainees, five trainees each had five pigs for EUS-CDS attempts.

The pigs were placed under general anesthesia using intravenous propofol (1 mL/kg) and intubated endotracheally using a single repeat dosing regimen to maintain anesthesia. During the operation, pigs were placed in the left lateral position and fixed with a mouthpiece. Pigs in the control and experimental groups were sacrificed at 7 and 14 days, respectively, after endoscopic operation. Pigs used for trainees training were also sacrificed after 14 days.

This animal study was approved by the Institutional Review Board and Ethics Committee of Shengjing Hospital of China Medical University (Approval No. 2021PS149K).

### 2.2 Equipment

The equipment used included a linear array echoendoscope (EG3870UT; Pentax, Tokyo, Japan) in combination with an ultrasound scanner (EUB 6500; Hitachi, Tokyo, Japan). Metal clips (ROCC-D-26-195, Micro-Tech/Nan Jing Co, Ltd, Nanjing, China) were used to clip the Vater’s ampulla. The metal clip is rotatable and can be opened and closed repeatedly. A 19-gauge needle (EUS N-19-T; Wilson-Cook Medical, Winston-Salem, NC, USA) was used for puncture. A 0.035-inch guidewire (Jagwire; Boston-Scientific, Natick, MA, USA) was used for guidance. A cystotome (6 Fr; Wilson-Cook Medical, Winston-Salem, NC, USA) was used to dilate the fistula. A fully covered self-expandable metal stent (60 mm/10 mm; Micro-Tech/Nan Jing Co, Ltd) or an electrocautery-enhanced lumen-apposing metal stent (15 mm/6 mm; Micro-Tech/Nan Jing Co, Ltd) was used for biliary drainage.

### 2.3 Standardized EUS-CDS Procedure

#### 2.3.1 Biliary tract dilation porcine model construction

A linear ultrasound endoscope was guided into the stomach and duodenum to scan the diameter of extrahepatic bile duct. Thereafter, we switched to a high-definition gastroscope that was guided into the duodenum to place metal clips for Vater’s ampulla ligation according to the treatment protocol (**Figures 1B-E**). Endoscopic ultrasonography was used to observe the widest extrahepatic bile duct diameter. The endpoint of the model construction was a greater than 1 cm diameter of the extrahepatic bile duct (**Figures 1F-I**).

#### 2.3.2 EUS-CDS procedures

**Figure 2:**
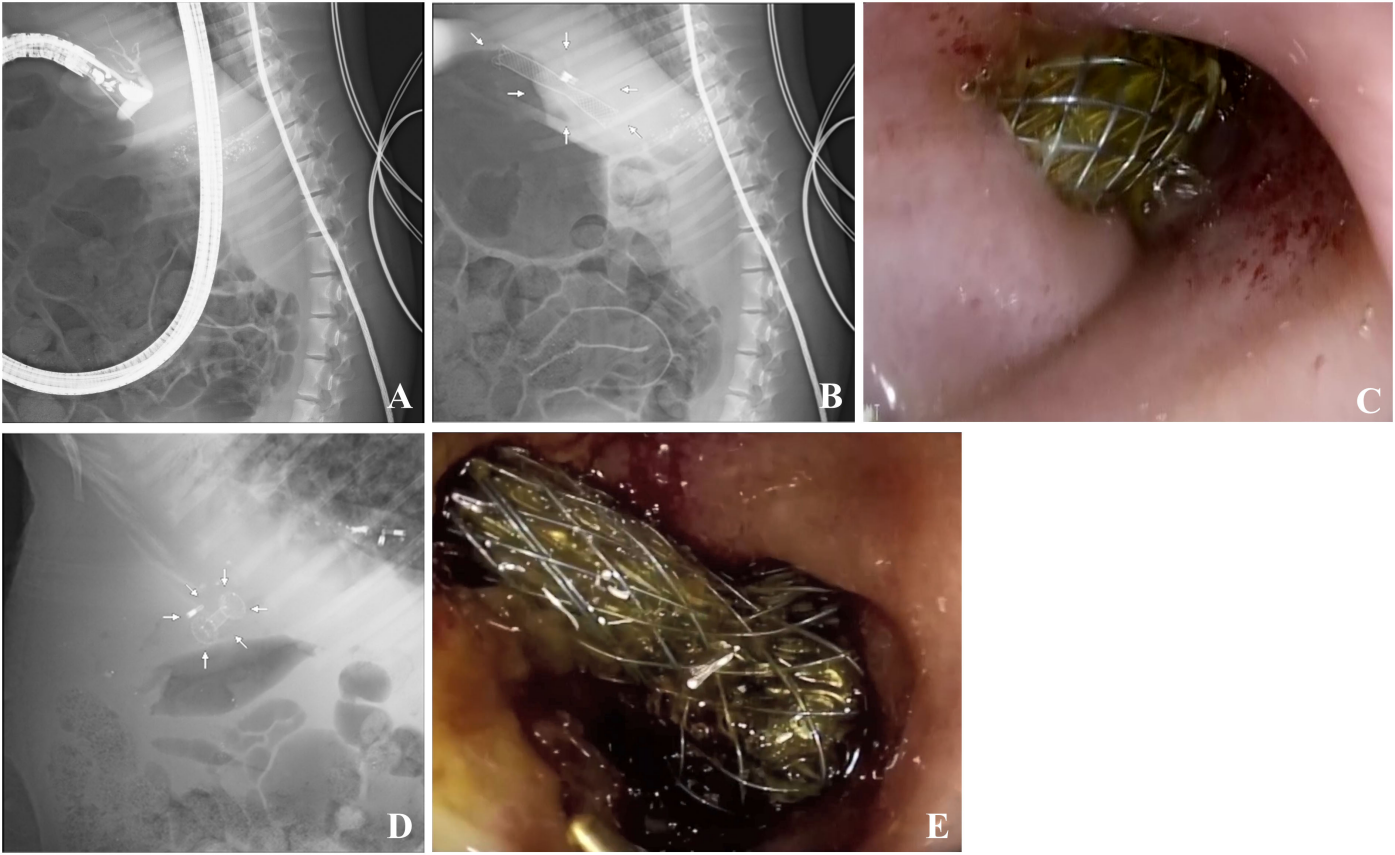
Extrahepatic bile duct puncture and ECE-LAMS/SEMS in a minipig model. **(A)** Extrahepatic bile duct puncture under fluoroscopic view. **(B)** Fluoroscopic image after stent deployment in ECE-LAMS (arrows). **(C)** Gastroscopic image after stent deployment in ECE-LAMS. **(D)** Fluoroscopic image after stent deployment (arrows) in SEMS. **(E)** Gastroscopic image after stent deployment in SEMS.

An experienced endoscopist performed EUS-CDS on pigs with an extrahepatic bile duct diameter wider than 1 cm. First, an ultrasound endoscope was advanced into the duodenum. Color doppler flow imaging was applied to avoid blood vessels. The CBD was punctured using a 19-gauge needle (**Figure 2A**). The bile was aspirated and the contrast agent was injected to confirm the CBD. Next, we replaced the 19-gauge needle with a guidewire and coiled it into the CBD.

The ECE-LAMS was incised layer by layer along the guidewire in the power-cut mode of the electrical surgical workstation. With the assistance of the assistant physician, distal end of the stent was released in the CBD. Subsequently, we continued to release the remaining part of the stent within the working channel of the EUS and slowly withdrew the endoscope. At this point, we could see the other end of the stent opening at the duodenum, and bile was flowing through the stent (**Figures 2B, C**).

As for the SEMS group, a cystotome was used to cut the duodenal and CBD walls along the guidewire to create a fistula. Lastly, a fully covered SEMS implant was guided into the CBD to replace the cystotome, and the stent was released. In the entire process mentioned above, guidewire replacement was of utmost importance (**Figures 2D, E**).

Finally, to confirm the success of the operation, the open end of the stent was flushed with saline under the field of a high-definition endoscope, and bile could be seen to be freely discharged from the visible segment of the stent in the duodenum. The primary endpoint of EUS-CDS was the ability to complete the above process, with the secondary endpoint being a decrease of more than 50% in total bilirubin over the course of a week.

#### 2.3.3 Validation of EUS-CDS training effect

To further validate this animal model for clinician training in EUS-CDS, we selected five endoscopists to learn and practice EUS-CDS on our 25 successful porcine models. They all have more than 5 years of experience in endoscopy and completed at least 60 cases of EUS interventional treatment each year, but have no experience in EUS-BD. The EUS-CDS learning materials and video materials were distributed to participants, and then they were asked to observe the EUS-CDS complete animal experiment 3 times. Next, they implemented EUS-CDS directly under supervision. Each participants had 5 chances to perform EUS-CDS on porcine models, and the procedures were fixed as ECE-LAMS. Mangiavillano et al. showed that LAMS has high technical and clinical success rates for various interventional endoscopic ultrasound procedures (11). The main scoring criteria were intraoperative performance, including CBD localization, fine needle aspiration of the CBD, guidewire replacement, fistula expansion, stent release, and bile outflow through the stent, with a total of six parts. The supervisor used a global rating scale (4-point scale) to give a comprehensive score to each part. 1 point was given for those who could not complete the whole process and needed the supervisor to take it over, 2 points for more oral guidance, 3 points for less, and 4 points for being able to complete the whole process independently (**Table 1**).

**Table 1:** Superiors’ assessment scoring table and the results of the 3rd exercise.

Trainee	CBD localization	fine needle aspiration of the CBD	guidewire replacement	fistula expansion	stent release	bile outflow through the stent	Total score
**1**	3	3	1	3	2	3	15
**2**	4	3	2	2	3	4	18
**3**	4	3	1	2	3	4	17
**4**	4	3	2	3	2	2	16
**5**	4	3	3	2	4	4	20

### 2.4 Statistical analysis

Results are reported as means ± standard deviations. All statistical analyses were performed by using GraphPad Prism (version 9.0; GraphPad software, La Jolla, California). We used student’s T-test for comparison between two groups, and one-way repeated-measure analysis of variance with Greenhouse-Geisser Correction for comparison between multiple groups. *P <*0.05 was considered statistically significant.

## 3 Results

**Figure 3:**
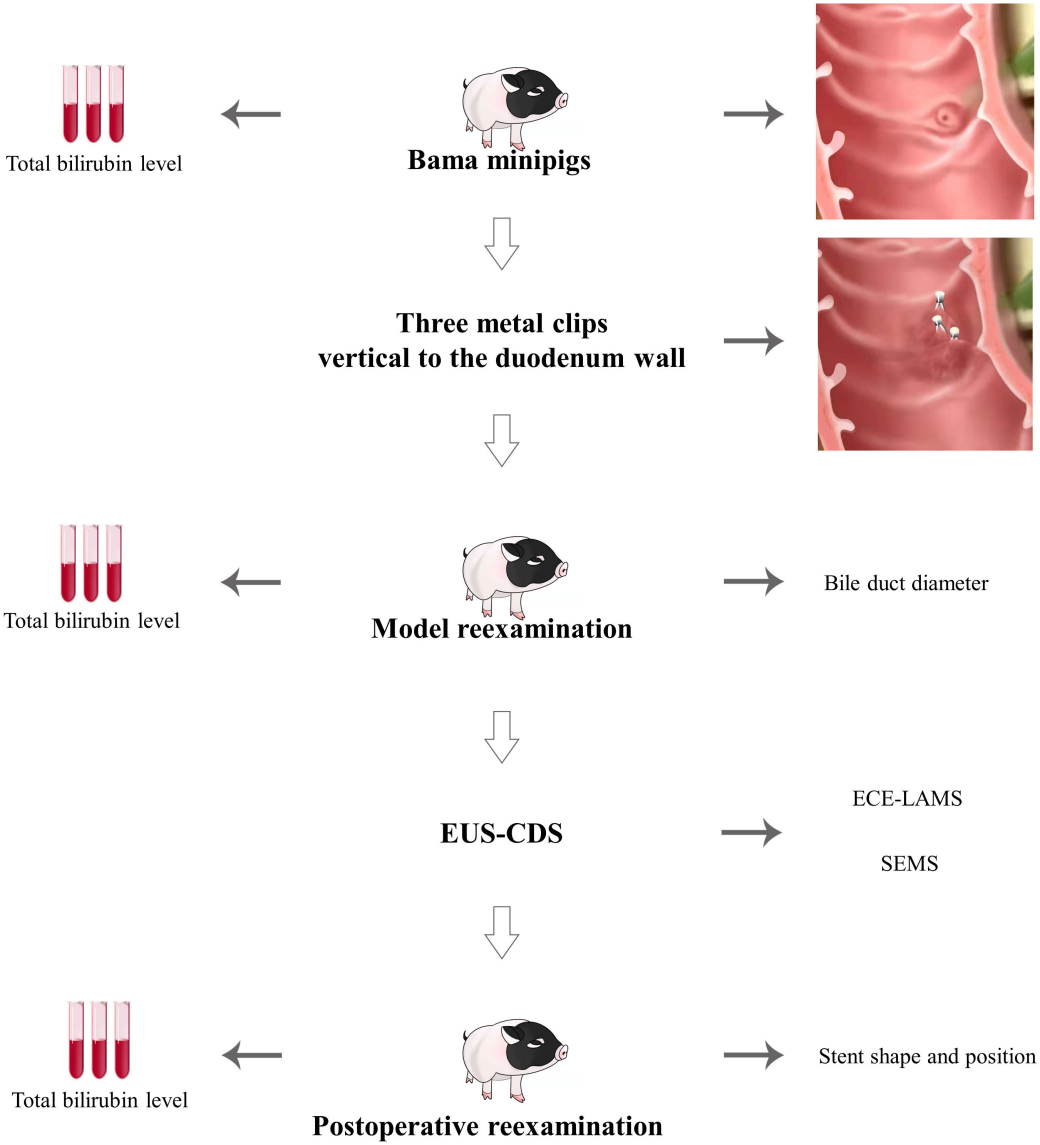
The overview of the pig bile duct dilation model and EUS-CDS.

In standardized EUS-CDS process construction, the diameter of extrahepatic bile duct in nine pigs was 0.30 ± 0.15 cm before operation. In Group A, there was no significant dilation 1 week after intermittent observation. There was no significant difference between preoperative and postoperative bilirubin changes and common bile duct diameter changes. In Group B, after clipping Vater’s ampulla, five pigs had extrahepatic bile ducts expanded to more than 1 cm in 24 h, whereas that of one pig expanded to more than 1 cm in 48 h. Compared with 24 hours before operation, the increase of bilirubin and the enlargement of the inner diameter of CBD were obvious, and the differences were statistically significant (*P <*0.05) (**Table 2**). The most noticeable dilatation point was along the middle part of the extrahepatic bile duct in six pigs; therefore, the middle part was selected as the CBD puncture site. The six pigs in Group B were randomly subjected to EUS-CDS via ECE-LAMS or SEMS, with three pigs for each stent type (**Figure 3**). All the operations were successful, and postoperative recovery was good, with no stents shifting or falling off.

**Table 2:** Total bilirubin level and bile duct diameter in the control (Group A) and experimental groups (Group B).

		Group A (n=3)	Group B (n=6)
**Total bilirubin level** **(μmol/L)**	**Before**	1.90 ± 1.51	0.82 ± 0.16
	**After 24h (before CDS)**	6.10 ± 7.40	27.67 ± 18.03
	***p*-Value**	0.343	0.015
		
	**1 week after CDS**		1.04 ± 0.56
	***p*-Value**		0.022
	**2 weeks after CDS**		0.58 ± 0.22
	***p*-Value**		0.020
**Bile duct diameter** **(mm)**	**Before**	2.77 ± 1.06	3.13 ± 1.79
	**After 24h (before CDS)**	3.12 ± 0.75	10.65 ± 2.60
	***p*-Value**	0.420	<0.001

Values are means ± standard deviations.

### 3.1 Three metal clips perpendicular to the duodenal wall is proposed as the standard for the biliary tract dilation model

In Group A, the metal clip did not fall off within a week after clipping Vater’s ampulla. Under the gastroscope, Vater’s ampulla appeared swollen, and a small amount of bile still entered the duodenum through Vater’s ampulla. The widest extrahepatic bile duct diameter in Group A was no more than 0.5 cm. Furthermore, there were no significant change in the total bilirubin level. In Group B, the inner diameter of the middle extrahepatic bile duct of five pigs expanded to more than 1 cm in 24 h post-operation. Although there is still no gold standard for this indicator, it is generally believed that when the diameter of the extrahepatic bile duct is greater than 1 cm, the success rate of stent implantation is higher. The total bilirubin level in Group B increased with increasing ampullary clipping time.

### 3.2 Proposed model is effective for EUS-CDS training and practice

None of the pigs showed any adverse reactions during the operation. During the experiment, bile leakage, bleeding, perforation, peritoneal effusion, stent displacement, or fall-off were not observed. However, we observed that after dissection in one pig with SEMS, a large amount of food remained in the stent, and the biliary tract system expanded more than normal. Total bilirubin in six pigs from Group B dropped to normal levels 1 week after stenting. The total bilirubin of the reflux pig in the SEMS group increased 2 weeks after stenting, and it was higher than that at day 1 after metal clip implantation. None of the pigs received reintervention treatment.

**Figure 4:**
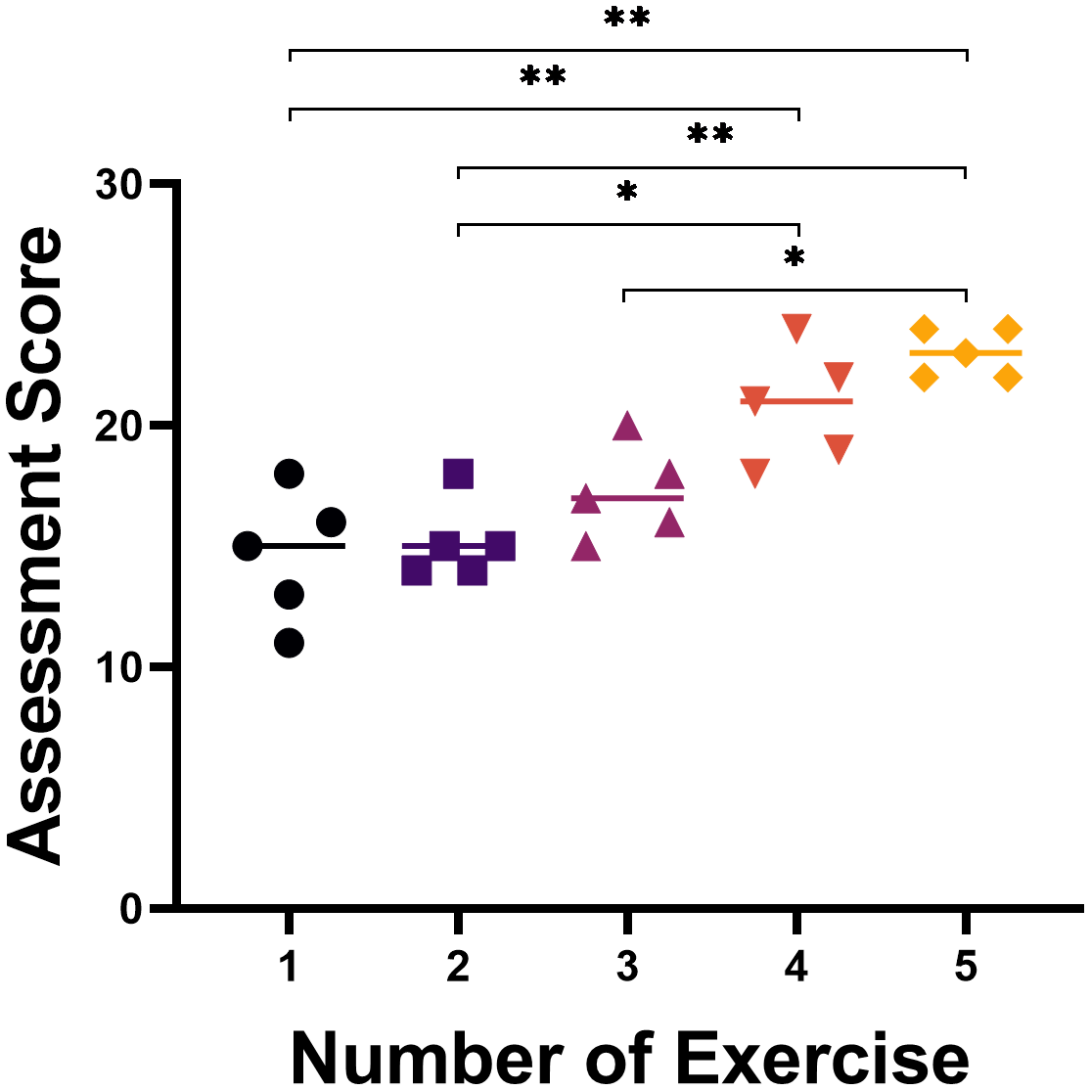
The learning curve of five trainees practicing EUS-CDS on porcine models. After the third training, the data showed a significant difference. *, *p*<0.05; **, *p*<0.01.

The learning curve came from superiors’ assessment scores and was plotted (**Figure 4**). The result showed that the scores of five trainees at different number of exercises were different (P<0.05). Compared with session 1, there was no significant difference between training sessions 2 and 3 (P > 0.05), but a significant difference between 4 and 5 (P < 0.01); compared with session 2, there was no significant difference for session 3 (P > 0.05), but significant differences for 4 (P < 0.05), 5 (P < 0.01); meanwhile, there was a significant difference between 3 and 5 (P<0.05). We found that, after 3 surgical exercises in animal models, the score has significant differences. All five participants were able to independently complete EUS-CDS on the porcine model after the training. Proficiency increases with the number of training sessions.

## 4 Discussion

Obstructive jaundice is a common clinical symptom, and the primary task of treatment is to remove obstruction as soon as possible, regardless of it being benign or malignant (12, 13). ERCP is the first choice to remove the obstruction. EUS-BD is an alternative drainage method when ERCP cannot be performed or fails (12, 14). According to recent research, the technical success rate of EUS-BD is more than 90% (15). EUS-BD intracavitary bile drainage pathways include hepaticogastrostomy (HGS) and CDS (12, 16). Here, we described the CDS method in detail and verify its training effect, and provided a good animal learning model for clinically novel and technically difficult endoscopic procedures.

With the increasing application of EUS-CDS, an appropriate animal model is urgently needed to train endoscopists to shorten the time to proficiency. Rimbas et al. showed that through hands-on training courses and training models, less experienced endoscopists could perform EUS-BD with CBD dilation close to 12 mm (17). Therefore, proper extrahepatic bile duct dilation is a prerequisite for EUS-CDS. Previously, Dhir et al. designed and improved a 3D printed digestive system model for endoscopists to practice EUS-BD (18, 19). This model could indeed help improve teaching and practice-related operating techniques, but it could not simulate the actual surgical environment. For instance, adverse events and needle penetration could hardly be simulated in the *ex vivo* model. Therefore, more scholars are focusing on the porcine model, which provides excellent tactile feedback and a high level of realism. To this end, Ligresti et al. used temperature-controlled endobiliary radiofrequency ablation (EB-RFA) to construct a biliary dilation porcine model (20). However, they operated on only one pig and did not define a standard procedure. Oh et al. used the same approach to model in seven pigs (21). However, inappropriate RFA action time and power selection may lead to the risk of bile duct perforation. Moreover, the operation was difficult, and it took a long time to construct a viable model. And since many centers do not have EB-RFA equipment, it may take longer for endoscopists to master this technique. Minaga et al. used endoscopic clips to ligate Vater’s ampulla and carried out an EUS-CDS stent study on the dilated common bile duct (10). Minaga et al. used multiple endoscopic clips to ligate each pig’s ampulla and using the newly designed metal stent with a thin delivery system to carried out EUS-BD (22). However, their research did not describe the modeling steps in detail, nor did they clarify the ligation method, changing CBD trend, and the corresponding change in total bilirubin. In addition, in their study, the diameter of CBD was up to 9 mm, which was not feasible for stents with a diameter greater than 10 mm, while in our study, the diameter of CBD was all greater than 10 mm. Studies have shown that over-the-scope clip (OTSC) clips the tissue more tightly than ordinary endoscopic clip (23). During the experiment, we also tried OTSC. In addition to the problem that the clip itself is expensive, it occupied a large space in the pig duodenum, which affected the operation. At the same time, the tolerance of pigs was not good, and it was easy to die after surgery. In summary, the construction of an *in vivo* CBD expansion model mainly adopts CBD ligation, shearing, or RFA. However, EB- RFA equipment and OTSC are not available in many endoscopy centers, and they are expensive, complicated to operate, and high cost of modeling.

In our study, two different clipping methods were used to establish a CBD expansion model and it was successfully constructed in Group B. In the course of our research, we attempted to clip Vater’s ampulla using two metal clips perpendicular to the duodenal wall; unfortunately, this approach did not guarantee successful modeling in every pig. Finally, we found that using three metal clips gave the best outcome. Concurrently, we found that in Group B, the most noticeable dilatation of CBD was in the middle segment, whereas that at both ends was not apparent. Nevertheless, the mechanism of this phenomenon is still under exploration. After the model was established, we performed EUS-CDS on six pigs and the stents did not shift or fall off, and the total bilirubin of the experimental animals dropped to close to the preoperative level. Therefore, we believe that the bile duct dilatation model is suitable for follow-up EUS-CDS surgery teaching and practice. In addition, this modeling method has elementary equipment requirements and no technical difficulties.

The complexity of EUS-CDS requires specialized skills in EUS interventional treatment and stent release. The learning curve of EUS-CDS is unknown and there are limited learning data for other therapeutic EUS. Practice is important, so we improve the surgical skills of endoscopists by providing preliminary preparation for EUS-CDS by constructing a suitable animal model. A retrospective analysis by Jovani et al. concluded that EUS-guided gastroenterostomy requires 25 maneuvers to achieve proficiency and 40 maneuvers to achieve mastery (24). Tyberg et al. found in a prospective study over six years that endoscopists with EUS-BD experience can shorten the procedure time and accelerate the acquisition of this skill with consecutive cases (25). Prospectively collected data by Oh et al. found that the operative time and the incidence of adverse events decreased after EUS-HGS 24 times (26). Therefore, we hope to shorten the time to master EUS-CDS skills by providing animal models that meet clinical needs for EUS-CDS training.

There are still some limitations in our study. First, our research was a small-sample animal experiment. Second, all surgical operations were performed independently by the same experienced endoscopist. This may be a potential bias to our study. Third, when verifying the training effect of our model, only 5 endoscopists and the number of participants was small. Finally, the porcine model is acute dilation, mainly used for personnel training or equipment development, etc. However, it cannot be used to study chronic pathophysiological changes, and each animal was sacrificed after 2 weeks of observation, so it may only show a short-term outcome. Therefore, to further evaluate the effectiveness of our animal model, more rigorous animal experiments with large samples and longer observation periods are required.

## 5 Conclusion

In summary, using three metal clips perpendicular to the duodenal wall for clipping Vater’s ampulla to construct a CBD dilation porcine model is a simple, stable, and effective endoscopic method. The effect of the model constructed by this method for EUS-CDS training is significant. Moreover, the model could also be used for further investigations of EUS-CDS.

## Data availability statement

The original contributions presented in the study are included in the article/supplementary material. Further inquiries can be directed to the corresponding authors.

## Ethics statement

The animal study was approved by the Institutional Review Board and Ethics Committee of Shengjing Hospital of China Medical University. The study was conducted in accordance with the local legislation and institutional requirements.

## Author contributions

XZ and NG completed animal experiments. XZ and ZL generated the figures and wrote the manuscript; WM and ShiS contributed to the writing of the manuscript; SiS and NG designed the aim of the editorial and revised the manuscript. All authors contributed to the article and approved the submitted version.
